# Patient experiences of crisis home treatment teams: a systematic review and thematic synthesis

**DOI:** 10.1007/s00127-025-02830-6

**Published:** 2025-02-17

**Authors:** Jialin Yang, Naomi Glover, Lisa Wood

**Affiliations:** Division of Psychiatry, University College London, 149 Tottenham Court Road, London, W1T 7NF UK

**Keywords:** Crisis home treatment teams, Patient experiences, Qualitative studies

## Abstract

**Purpose:**

Crisis home treatment teams (CHTTs) provide rapid at-home assessments and crisis support to individuals experiencing mental health crises. Exploring patient feedback on CHTTs can provide policymakers and service planners useful insight regarding service enhancements and improvements in care quality. The current systematic review aims to explore patient experiences on CHTTs, to inform policy decision-making and care quality.

**Method:**

The current review synthesised ten eligible qualitative studies (from Medline, PsycINFO, Embase and CINAHL) on patient experiences of CHTTs using thematic synthesis. Adult patients with a past or current experience of CHTTs, as well as a mental health diagnosis were included in the review.

**Results:**

The current review revealed that patients valued the rapid accessibility of services and positive characteristics of staff that contributed to cultivating strong therapeutic relationships. Patients also appreciated having equal power in treatment decision-making. However, concerns were raised regarding staff timekeeping, receiving generic treatment not well-tailored to patient’s unique circumstances, and inconsistencies in continuity of service delivery and smooth transition to other services.

**Conclusions:**

Patient feedback on service improvements are useful for service planners and policymakers to improve CHTT services. Based on the results of this study, the importance of staff timeliness, having a smooth transition during the end of care, and tailored staff training for various demographics can improve CHTT service quality and delivery. Further qualitative research is needed to gain a comprehensive understanding of patient needs and experiences in various regions and demographics.

**Supplementary Information:**

The online version contains supplementary material available at 10.1007/s00127-025-02830-6.

## Introduction

Crisis home treatment teams (CHTTs) provide rapid assessments for individuals experiencing mental health crises and offers at-home treatments as an alternative to usual inpatient treatment [1]. CHTTs offer support to individuals with a high risk of acute inpatient admissions, or those requiring intense short-term or long-term care, or individuals recently discharged from inpatient units [1]. Today, progressive steps have been taken regarding the implementation of CHTTs. The re-configuration of acute mental health services in Australia and North America influenced service implementation in the UK and other community-based initiatives in Western Europe [2]. For instance, the development of CHTTs in the UK has gradually increased from having only 35 CHTTs in 2000 to having 198 adult CHTTs in 2018 [3]. The UK National Health System (NHS) has increased its investment for CHTTs, from £38 million in 2002 to £268 million in 2011 [4]. More recently, the UK Government injected another £150 million into improving mental health crisis services up to 2025. In 2012, the Royal College of Psychiatrists established the Home Treatment Accreditation Scheme (HTAS) to facilitate quality improvements on delivering CHTT services. Today, the HTAS is known as the Quality Network for Crisis Resolution and Home Treatment Teams (QN-CRHTT). The QN-CRHTT also provide protocols to protect patient safety and standardises treatment expectations for CHTT staff [5].

Despite the gradual shift from in-hospital treatments to CHTTs for mental health crises, the effectiveness of CHTTs gatekeeping and reducing inpatient admissions remains inconsistent [6]. A large RCT study that compared the presentation of crises before and after a CHTT service was introduced found a large decrease in inpatient admissions. In six weeks, the number of hospital admissions reduced from 71 to 49% after a crisis service was introduced [7]. However, a survey by Onyett [8] found inconsistencies in CHTT service delivery among 243 CHTTs in England. Specifically, a third of the teams were not involved in gatekeeping from inpatient admissions, and just over half of services offered a 24/7 visiting service [8]. The cost of CHTTs compared to inpatient treatment is under-researched and remains inconclusive. A systematic review revealed that CHTTs were in fact cheaper compared to inpatient care. However, costs are highly dependent on the local catchment area, for example, the location of services, the availability of hospital beds, and the number of people receiving care from CHTTs [9].

Patient experiences on CHTTs also have mixed evidence. Some studies have revealed that families, including patients and carers, were more satisfied with CHTTs than inpatient care and provided more positive feedback on CHTT services [10]. Previous research examining patient experiences stated that CHTTs provided practical support in daily living, reassurance, accessibility, and flexibility from staff [11]. Themes such as being treated as a normal human being through attentive listening and respect, being able to deal with crises independently in daily life were also important to patients [12]. However, patients also reported negative experiences that hindered their recovery process. For instance, the lack of continuity of care and follow-ups [11], issues with accessing care, and services having a narrow focus on medication rather providing practical support [13].

Patient satisfaction is not discussed extensively in research as many studies examine CHTTs and reductions in inpatient admission rates. Therefore, examining patient experiences and insights are vital for CHTTs to improve quality of care, service development and implementation [14]. However, patient satisfaction, continuity of care, accessibility of care and other aspects of patient experiences are under-researched, with most existing research conducted in the UK and Europe [15], indicating a research gap that needs to be examined further. Patient feedback can facilitate CHTTs to be more tailored to patient needs and implement better quality mental health care in times of crises [16].

The aim of this systematic review is to examine existing qualitative literature of patient experiences on CHTTs, contribute to existing literature and synthesise important feedback regarding beneficial and challenging aspects of CHTTs.

## Methods

### Study protocol

The research protocol of this systematic review was prospectively registered on the international database for registered systematic reviews, PROSPERO (ID: CRD42023440564) on June 29th, 2023.

### Eligibility criteria

The systematic review included studies with (a) patients aged 18 and above, (b) patients with a past or current experience of using CHTTs with no restrictions to the duration of treatment, and (c) qualitative studies including interviews and focus groups, that are (d) published in or translated to English, which (e) examine aspects of patient experiences included (but not limited to) the satisfaction of care, accessibility of services, quality of care, consistency of care, and costs of care received in CHTT. The eligibility of studies was determined based on the following CHTT definition: a crisis services consisting of a multidisciplinary team that a) conducts assessments for all service-users considered for inpatient admissions; b) provides home treatment and frequent visits (at least daily) within service-users’ homes; c) delivers home treatment until crises are resolved and sign-posts service-users to other services according to their needs; and d) facilitates the transition from inpatient units by offering intensive home-based treatment [17]. Mixed methods papers consisting of both qualitative and quantitative results were also included, but only qualitative results were analysed.

The review excluded studies with (a) patients under the age of 18, (b) patients using other types of crisis services such as inpatient admissions or crisis houses, and (c) patients with a primary diagnosis of (i) learning disabilities, (ii) substance misuse, or (iii) any organic impairments. The decision to exclude participants with a primary diagnosis of learning disabilities, substance misuse or organic impairments is deliberate, as the review aims to specifically concentrate on mental health crises.

### Search strategy

Searches were conducted on four electronic databases (Medline, PsycINFO, Embase via Ovid and CINAHL via EBSCOhost). Searches on Google Scholar were also undertaken to identify potential grey literature that may not be included in the four databases. Due to patient experiences of CHTTs being under-researched, this review included studies published in any year. This allows the researchers to gain a comprehensive scope the evolving insights and perspectives of patients as CHTTs have progressed over time. The initial search was conducted by the first author (JY) in June 2023. An updated search was conducted in September 2024, to identify potential new publications for inclusion but no further eligible studies were identified. The search consisted of three key concepts: Patient experiences, CHTTs, and qualitative studies. The searches included relevant search terms and subject headings for patient experiences (e.g. patient, consumer satisfaction, experience), CHTTs (crisis intervention, home treatment team, home based treatment teams), and qualitative research (qualitative, interview, focus groups). All three concepts were searched through utilising a combination of subject headings and text-words. The complete search strategy and search dates for all four databases are available in the supplementary material (Appendix 1).

### Selection process

All studies were uploaded onto the software EndNote 20 [18] for deduplication and then imported onto Rayyan for screening [19]. The titles and abstracts of studies were screened independently by JY, and a second reviewer, who independently screened 20% of the total studies. The consensus between JY and the second reviewer established a substantial inter-rater reliability measured by Cohen’s Kappa value (*k* = 0.748), suggesting a strong agreement between the raters [20]. Then, full-text screening was conducted for all eligible studies by JY and discussed uncertainties with LW. There was no missing information in the included studies, so no authors were contacted for additional information.

### Data extraction and synthesis

All eligible full texts’ data were extracted into a table summary (see Table 1) by JY on the 28^th of^ July 2023. Study authors, year of publication, settings of the study, study sample, study design, length/duration of received treatment, outcome measure(s)/follow-up measure(s), and main outcomes were documented in the table. Then, the qualitative results sections of the included studies were imported into the qualitative data analysis software NVivo 12 for thematic synthesis.

**Table 1:** Key characteristics of included studies

Study	Setting of study	Sample	Study Design	Length/Duration of Treatment	Outcome Measure(s) & Follow-up measures	Main Results
Carpenter & Tracy (2015): Home treatment teams: what should they do? A qualitative study on patient opinions	A Home Treatment Team (HTT) in Bromley, South East London	*Patients (recently discharged from the HTT)*: *N* = 10 *Age*: 25–55 Male: *N* = 4 Female: *N* = 6	Semi-structured interviews; interview duration ranged from 10 to 50 min. *Data analysis*: Thematic analysis	Duration ranged from 2 to 33 days of receiving treatment from HHT.	13-item semi-structured; explored opinions of ex-CRT patients on the aspects the services provide or not provide	*Six main themes*: time, ending of care, talking, alternative to hospital admissions, staff characteristics, improvement of mental health Patients highlighted the importance of rapidity of receiving care after crisis, talking to clinicians of HTTs. Staff being late for appointments and timing were points of concern.
Giménez-Díez et al. (2019): Treating mental health crises at home: Patient satisfaction with home nursing care	Crisis Resolution and Home Treatment Team (CRHTT) service in Hospital de la Santa Creu I Sant Pau (UHPAD), Catalonia, Spain.	*Patients*: *N* = 20 Male: *N* = 7 Female: *N* = 13	*Mixed methods design*: quantitative survey and qualitative semi-structured interviews; interview duration was one hour. *Data analysis*: A conceptual framework with a phenomenological focus	Patients (and carers associated with one patient) who received treatment for two months in the UHPAD team.	*Qualitative measures*: Semi-structured interviews that explored aspects of comfort, technique, communication during care	Patients and carers of patients all expressed high levels of satisfaction of treatment. The nurses were seen as professional, accompanying, supportive and understanding. Patients felt that daily visits contributed to the continuity of care, creating a strong relationship between the patient and nurse. Other important aspects included availability of nurses, patients being seen as “normal” and felt less stigmatising.
Hopkins & Niemiec (2007): Mental health crisis at home: patient perspectives on what helps and what hinders	The Newcastle Crisis Assessment and Treatment Service (CATS) in Newcastle upon Tyne (Northeast England). The Newcastle CATS serves an adult population of 450,000 in urban areas of Newcastle and North Tyneside Multidisciplinary team, operating 24/7, with main roles of crisis triage, assessment and resolution through home-based/community treatment, gate-keeps inpatient admissions.	*Patients*: *N* = 76	Two-staged modified Delphi study and semi-structured interviews and questionnaires *Data analysis*: Thematic analysis	Patients who had been receiving at-home treatment during its initial 16 months.	*Measured outcomes*: Accessibility Availability Consistency Quality Choice /negotiation Communication Changes and endings	*Main themes*: Patients believed that accessing treatment easily and quickly at the start of crisis is important. Home treatment was available to them in a timely manner. Patients highlighted the importance of being listened to respectfully, and clinicians being calm and friendly. Patients expressed good communication in all aspects of care. A fully informed and negotiated ending, with smooth transition to other services is important.
Hubbeling & Bertram (2014): Hope, happiness and home treatment: a study into patient satisfaction with being treated at home	Wandsworth Crisis and Home Treatment Team (London, UK) Offering services such as home visits (from 9am to 9pm) and telephone hotline outside working hours.	*Patients*: *N* = 117	*Qualitative study*: Questionnaire developed by authors (with open questions) *Data analysis*: content analysis	Received/receiving treatment in the service for up to 4 weeks	Authors developed own questionnaire, based on the Care Quality Commission’s National Survey; includes open questions	Patient satisfaction was higher with receiving various elements of care. Some said it was good receiving treatment at home, but patients also said they were seen by too many people, which had conflicting views between patients. Patients also recommended that services should go to appointments more on time and have a written care plan provided to them.
Karlsson, Borg & Kim (16): From good intentions to real life: introducing crisis resolution teams in Norway	A local Crisis Resolution Home Treatment Team near Oslo, Norway	*Patients*: *N* = 7 Male: *N* = 2 Female: *N* = 7	In-depth research interviews *Data analysis*: Hermeneutic content analysis	Patients in contact with local CRHT at least two times within the last year, and ended the contact within the last six months	Examined patient experiences of CRHTs, focusing on how they were treated by the team, solutions the team provided, supportiveness of the team (in comparison to inpatient treatment they received previously before CRHTs)	*Main themes*: Sense of control; patients experienced greater sense of control of their treatment, discovered new possibilities from their crises, experienced personal growth. Opportunities for participation; patients felt more secure and safe, and the treatment was seen as helpful. Being seen and listened to; patients expressed the importance of being seen and listened to, taken seriously, and being respected by the team.
Khalifeh et al. (2009): Home treatment as an alternative to hospital admission for mothers in a mental health crisis: a qualitative study	Crisis Resolution Teams (CRTs): Home treatment teams in Camden and Islington (London, UK)	*Patients (all females)*: *N* = 18 *Age*: 20–60 *Diagnosis*: Major Depression (*N* = 10) Bipolar Disorder (*N* = 6) Schizophrenia (*N* = 2)	Semi-structured interviews (over 6 months period) *Data analysis*: Content analysis	Patients receiving treatment by one CRT in the previous 18 months	Interviews explored patient’s experiences of home treatment, children’s experiences of home treatment, treatment preferences and unmet needs.	*Advantages of home treatment*: Receiving good quality care from CRTs and avoiding hospital admissions. Most patients with pasted experience of inpatient hospital treatments all preferred home treatment because they felt safer and better looked after. *Disadvantages of home treatment*: Difficulty in parenting during crisis, exposing children to destressing symptoms or behaviours, burdening their child(21) with caregiver responsibilities (Only adult data were analysed due to eligibility criteria)
Klevan. Karlsson & Ruud (2017): “At the extremities of life” – Patient experiences of helpful help mental health crises	Eight Crisis Resolution Teams (CRTs) located in Norway	*Patients*: *N* = 14 *Age*: 25–70 Male: *N* = 8 Female: *N* = 6	Semi-structured in-depth interviews; interview duration ranged from 90 to 120 min. *Data analysis*: Hermeneutic phenomenological approach	Patients who had received services from local CRTs within the last 3 months but no longer in treatment	Semi-structured in-depth interviews focusing on experiences of mental health crises, perceptions of helpfulness (lack thereof) of CRTs, and the amount of support offered during crises.	*Helpful CRT practices*: Recovery-oriented understanding of crisis, clinicians were attentive to people’s crisis and needs, established safety and strengthen patients’ sense of self. The urge for establishing safety and feeling safe was a recurring theme.
Morant et al. (2017): Crisis resolution and home treatment: stakeholders’ views on critical ingredients and implementation in England	NHS Mental Health Service Trusts with CHTT services in England; locations covered metropolitan, mixed and rural areas.	*Patients*: *N* = 42 Male: *N* = 14 Female: *N* = 28	Focus group and individual interviews *Data analysis*: Thematic analysis	Number of times using the CRT service: 1 to 10 + times	Topic guides on views of CRT services, access to and discharge from service, important aspects of CRT care, interface with other related services, recommendations for good practices, barriers and facilitators to achieve better practice. Areas of concern such as continuity of care, types of care were also explored.	*Main themes*: Providing rapid responses, frequent home visits were deemed important for patients and carers. Being accessible, reliable and flexibility in providing services were also valued. Emotion support and gate-keeping inpatient admissions were important aspects of the role of CRTs. Referral pathways, lack of staff continuity was seen as negative experiences of CRTs.
Nelson, Miller & Ashman (2016): ‘Dale’: an interpretative phenomenological analysis of a patient’s experience with a crisis resolution/home treatment team in the United Kingdom	A local Crisis Resolution and Home Treatment Team in the UK.	*Patients*: *N* = 1	Semi-structured interview; and retrospective narrative from patient ‘Dale’ *Data analysis*: Interpretative phenomenological analysis	Twenty years of therapy, unknow how long the patient has received treatment from CRHTTs.	Interviewed on experiences of using CRHT services.	Dale had a generally positive experience. He felt that he was supported practically, socially, psychologically and educationally by the staff. He built strong therapeutic relationships with the team. Staff of the team helped Dale build his resilience, however it was difficult in maintaining positive learning while in an episode of mental health crisis.
Rubio et al. (2021): Experiences of intensive home treatment for a mental health crisis during the perinatal period: A UK Qualitative study	Seven diverse NHS Healthcare providers across England; with diverse sociodemographic and diagnosis background	*Patients (all female)*: *N* = 15 *Age*: 19–29	Semi-structured interviews; interview duration was approximately one hour. *Data analysis*: Thematic analysis	Patients that accessed CRTs during or after their most recent pregnancy.	Explored patient experiences of accessing services, information, choice and decision-making, views of services, perceived gaps, communication between services, involvement of family members and therapeutic relationships.	*Main themes*: Frequent home visits and continuity of care, brief risk-focused support, knowledge and understanding of the perinatal context, and the choice of specialist service availability. Majority of women found the services to be intrusive, impersonal and not tailored to the perinatal context. Continuity of care was highly valued but disliked that the visits were conducted by different professionals every visit. Better tailored crisis care is needed in the context of perinatal women.

The thematic synthesis was conducted based on Thomas and Harden’s guidance [21]. First, JY conducted line-by-line coding and codes were generated for each study with no hierarchal structure. Then, JY grouped the codes into broader analytical themes to capture commonalities within the studies. The broader themes contained multiple sub-themes that addresses specific concepts relevant to each theme, which were agreed by the research team.

The analysis was conducted by JY, a research student with no prior experience in CHTTs and supervised by LW, a clinical psychologist and researcher with experience in qualitative research and working clinically in CHTTs, and NG, a clinical psychologist who has previous experience in acute services and in a community mental health team that frequently works alongside CHTTs. Reflexive notes were kept by JY and discussed with supervisor LW during the research process to mitigate potential researcher biases that could influence the interpretation of data, thereby contributing to a more rigorous and balanced evaluation of patient experiences.

### Quality appraisal

The current review utilised the Critical Appraisal Skills Programme (CASP) Qualitative Checklist (see supplementary material Appendix 2) to assess risk of bias for the included studies. The CASP Qualitative Checklist is widely recognised in qualitative research for assessing study quality in health-related fields and is endorsed by the Cochrane Qualitative and Implementation Methods Group [22]. The checklist consists of ten questions and is divided into three sections, each addressing a different area of qualitative research: aims and methods, results, and the value of research. The checklist is completed through selecting one of three options: ‘Yes’, ‘Can’t Tell’ or ‘No’ for each question. If ‘Yes’ is selected for the majority of the checklist, it indicates the study has a high-quality score. Contrarily, if ‘No’ is selected for the majority of the checklist, it suggests that the study has a low-quality score.

All risk of bias assessments were conducted by JY independently and the conclusions were cross verified by LW to ensure rigor and reliability. No studies were excluded based on the quality score.

## Results

### Search results

**Fig. 1:**
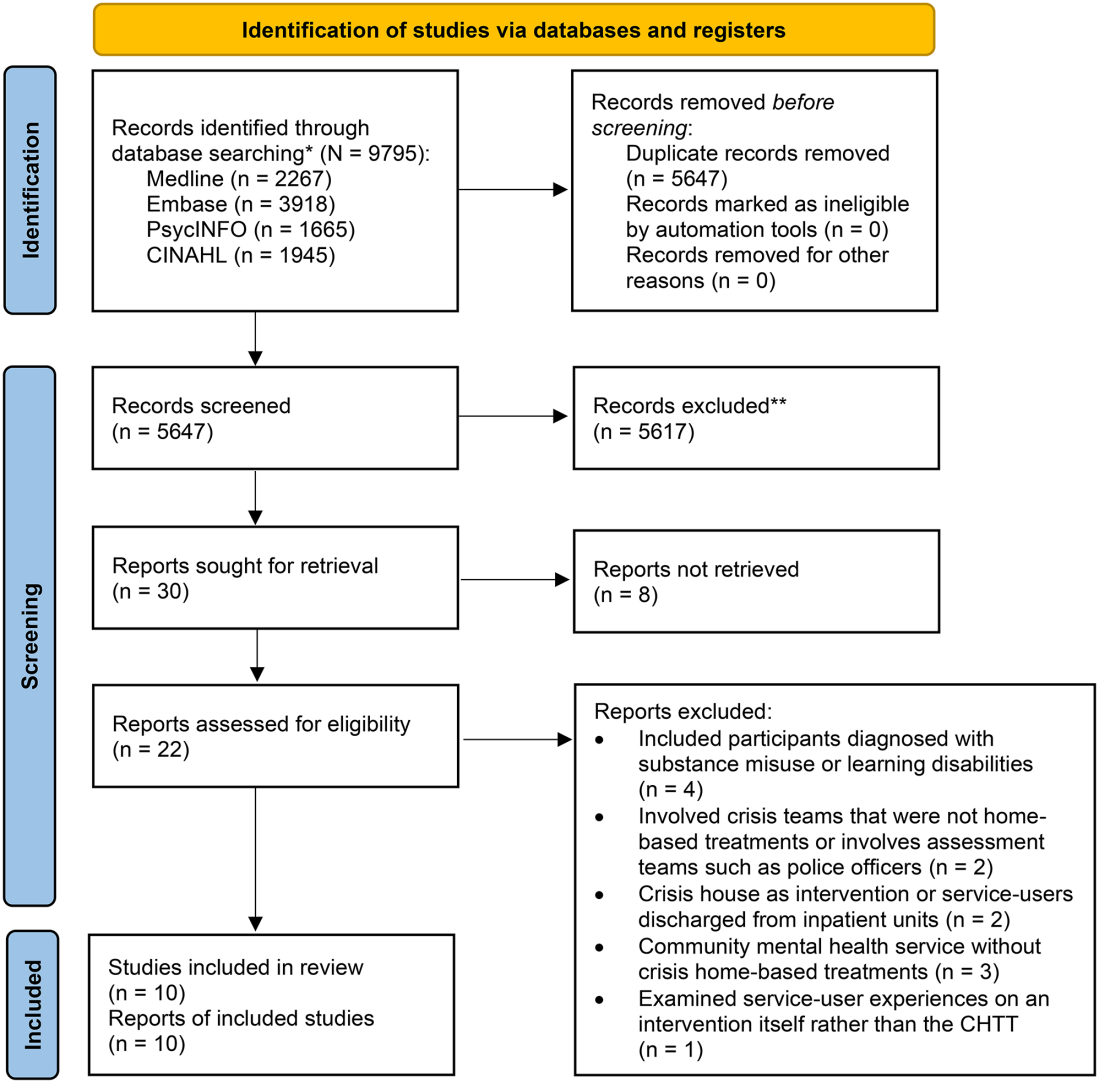
PRISMA diagram of study selection

Following deduplication, a total of 5150 studies yielded from the search. Subsequently, 5120 studies were excluded during the initial screening, and 30 studies were examined for full-text screening. After full-text screening, eight studies were excluded due to unavailability of full-texts, which were due to articles containing only conference abstracts. An additional 12 studies were also excluded because they included participants with a primary diagnosis of substance misuse or learning disabilities (*N* = 4), or examined crisis teams that were not CHTTs (*N* = 2), crisis houses (*N* = 1), patients discharged from inpatient units (*N* = 1), community mental health services excluding CHTT (*N* = 3), or examined patient experiences on a specific CHTT intervention rather than CHTTs as a service (*N* = 1). The final analysis included 10 studies. The updated search conducted in September 2024 yielded no new relevant papers for inclusion (see Fig. 1). No further relevant grey literature was identified on Google Scholar.

### Study characteristics

The total number of patients included was *n* = 320, with sample sizes ranging from *n* = 1 to *n* = 117 participants. There were seven studies conducted in England, of which three were based in London, one in Newcastle, and the remaining studies were conducted across various NHS Trusts across England. Additionally, three studies were based in Europe, comprising two studies in Norway and one in Spain. Most of the sample population were female (73% female, 27% male), with ages ranging from 19 to 70 years (gender data available in *n* = 8 studies). In the *n* = 3 studies that reported participant ethnicities, participants were mainly White British (67%), and the remaining participants were Black Caribbean (13%), Black African (4%) and Asian (16%). Eight studies utilised a semi-structured interview study design, and the remaining two adopted a mixed methods design with questionnaires that included open-ended questions that encouraged narrative responses. Interviews were conducted by researchers (*n* = 7), and mental health professionals in CHTTs (*n* = 3). Thematic analysis was the predominant method of analysis (*n* = 4) among the included studies. The complete summary of study characteristics is illustrated in Table 1.

All included studies were of high quality, with CASP scores ranging from 7 to 10, with 10 as the highest score. All studies consisted of clear aims and appropriate methodologies, such as semi-structured interviews and focus groups. The findings of all studies were detailed with relevant quotes and of suitable method of analyses (e.g. thematic synthesis or content analysis) and included valuable reflections on the findings that contribute to service improvements.

### Thematic synthesis

There were four main themes identified: accessibility of care, the importance of therapeutic relationships, user empowerment in care involvement, and the consistency and continuity of care (See Table 2).

**Table 2:** Summary of themes

Themes	References
Accessibility of Care	[13, 16, 23–30]
The importance of therapeutic relationships	[13, 16, 23–25, 27–30]
User empowerment in involvement of care	[13, 16, 23, 24, 27–30]
Consistency and continuity of care	[13, 23, 24, 26, 27, 29, 30]

### Accessibility of care

Numerous patients expressed the value of accessing services rapidly. The concept of CHTTs being an alternative to inpatient admission was appreciated in multiple studies [23].

#### An alternate to inpatient care

Patients frequently highlighted their preferences of CHTTs over inpatient care. Patients found that the familiarity of their home environment to be less stigmatising and more conducive to their crisis recovery. The convenience of receiving treatment at home was resonated positively among patients as it was a better experience than being treated in inpatient wards, with it resembling like a “German concentration camp” described by one patient [36]. The sense of safety and security of at-home treatments stood out as a significant aspect in patient experiences.

Help has become more available… Two professionals came into my home, took a lot of initiatives, listened to me…It was in contrast to my earlier experiences with acute ward at the hospital (Patient [16])

However, some patients preferred the choice of being admitted because of the difficulties in parenting during a crisis. Some patients felt that inpatient admissions were a temporary escape from their responsibilities at home, where they did not need to worry about burdening their carers or children with their crises.

You know, being looked after, not having worried about things, deadlines, or anything. I’m not burdening my family. You’re getting more of a respite when you’re in hospital (Patient [23])

#### Rapid and timely access to services

Having rapid access to care and receiving care in a timely manner was essential to patients. Services that provided 24/7 access and frequent visits were valued, as patients felt that the availability of services and frequent visits served as motivation for improvement [24], and a source of safety [25].

Despite the swift accessibility of services, a recurring concern patients raised was the unpredictability of time and punctuality of CHTT staff attendance [13, 23, 24, 26]. Patients described experiencing late arrivals from staff or unscheduled visits, which was a source of anxiety and uncertainty. This was especially apparent in mothers due to difficulties in juggling parenting responsibilities while receiving treatment at home.

They couldn’t tell me when they were going to come, and I had a newborn baby, and they would rock up whenever… There would be two or three of them and they would just get in the way… It was worse having them than not having them. (Patient [24]).

To address these recurring concerns, patients expressed that they would prefer more flexibility and involvement in scheduling appointments with CHTT staff [13]. Moreover, it would be more helpful if staff provided patients with appointment slots [26], so they can attend appointments at allocated times to prevent late or missed appointments.

### The importance of therapeutic relationships

Altruistic and positive interpersonal qualities of CHTT staff were greatly valued by patients, contributing to fostering meaningful therapeutic relationships with the staff. Aspects such as attentive listening, non-judgemental support, and friendliness were deemed as important.

#### Being treated as a “normal human being”

The importance of being treated like a “normal human” rather than a “set of symptoms” was crucial for crisis recovery for patients. Being non-judgemental, respectful, friendly, and using lay-audience language to communicate with patients were aspects that helped with patients’ recovery [27].

As I shook and cried in a chair, they sat on the floor in front of me, attentive and engaged. This was a great shift from the usual patient/professional power dynamic that I was used to. (Patient [28])

#### Feel listened to and reassured

Feeling heard and reassured by CHTT staff was also appreciated by patients, especially when sharing experiences of their crises. These aspects were crucial for forming trusting therapeutic relationships [16].

Just listening as well, listening to you going on… because you were telling them an awful lot about yourself and they’re not judging you… they never make you feel little. (Patient [13])

Staff being comfortable and supportive to patients helped them feel more reassured and understood, which increased their sense of safety and self-worth [13, 25, 29].

#### Feeling safe and supported

Moreover, being in a safe and well-supported environment was vital to forming caring relations [13, 16, 30]. Staff providing emotional support was deemed helpful and encouraging [24]. In a study conducted in Norway, CHTT staff offering small gestures such as going on a walk or holding patients’ hand were actions that was appreciated during times of crisis [25].

They would come and pick me up. I remember it felt so good, going down to the sea. Just that 1 hour… and we just sat there… chitchatting… I will never forget it. (Patient [25])

#### Impersonal service

Although many patients reported positive experiences with CHTT services, some patients expressed frustrations with impersonal and generic visits that lacked depth. Some felt that the teams were mainly focused on risk management and medication management [13]. Moreover, patients emphasised that staff showed little interest in their lives and lacked warmth, which hindered open communication, particularly in perinatal women fearing judgement or the possibility of having their child taken away [24].

… A lack of more meaningful support paradoxically meant some women were fearful of being honest with crisis team professions about their feelings… therefore less likely to engage or disclose relevant information… in this case resulted in hospitalisation of their babies taken away (Authors [24])

### User empowerment in involvement of care

Having equal power in the involvement of treatment between patients and CHTT staff were deemed important. The involvement of decision-making and having a choice in treatment options were essential for patients [16, 23, 24, 27, 28, 29].

#### Involved decision in care plans

One of the most valued aspects of CHTTs was the equal involvement of decisions in care and having a choice in treatment plans for some patients. Patients being treated at home gave them more sense of control over their treatment and have equal say with staff. For instance, having a sense of control included patients formulating new ideas and possibilities in a situation of crisis, which helped with their recovery.

The information provided… helped them make decisions regarding their health… to understand important concepts regarding the recovery process… respecting the patient as an expert regarding experience (Authors [29]

They explained what they could do… but asked me what I wanted to do. I had to make a decision. I was given both the power and responsibility to do that. (Patient [28]

While many patients appreciated having shared power in treatment decisions, some felt that they were incapable of planning while being in a state of crisis and preferred staff to make decisions on their behalf [27].

To me it wasn’t important to negotiate. I just took what was offered to me at the time. I was in too much of an upset state to negotiate anything… Negotiating my care wasn’t very important to me at that time. (Patient [27])

Moreover, some patients felt that care plans were not explicitly stated, which was deemed unhelpful because it caused uncertainty and triggered a sense of insecurity [25]. For future improvements, patients suggested that treatment plans should be clearly communicated, and patient preferences should be considered.

### Consistency and continuity of care

Lastly, having consistent service delivery and regular contact from staff was integral for crisis recovery. Patients felt that they were often seen by different staff members during their treatment [26, 27] and were given contradicting advice between staff. The inconsistent personnel and contradicting advice were a source of confusion and anxiety.

The least helpful thing, for me, was not knowing the person that’s coming through the door. It was hard for me to talk to a total stranger… because I don’t know you, I’m not going to talk to you… Unfamiliarity to me, it’s quite difficult… When I’m at the point where I need the crisis team I prefer to have somebody who’s familiar that I can have that continuity with (Patient [13])

Contrarily, while some patients felt the contradicting advice was frustrating, a minority of patients appreciated the differing advice from staff. Patients also reported that all the staff were up to date with their situation and ensured their support was as seamless as possible [13].

#### Transition to other services and end of treatment communication

Having a seamless transition to other services or a smooth end of treatment plan was commonly raised by patients. Some stated that it was an aspect they were least satisfied with. While many patients were content with the end of care communication from CHTT staff [13], others faced abrupt endings with no follow-up, and was left suspended between services [24].

Changes were fine… endings were a different story; I felt I wasn’t given enough information about what to do if I suddenly had another crisis… You felt a bit out in the cold. (Patient [27]

Having changes in personnel during visits and abruptly ending the treatment with no follow-ups may have negative impacts in recovery and managing future potential crises [24, 27]. During times of uncertainty, patients stressed the need for sufficient warning and information regarding treatment discharge. CHTT staff providing explanations and assistance in navigating other services were suggested recommendations [27].

## Discussion

The current systematic review examined patient experiences and preferences of CHTTs through various themes, unravelling both positive and negative aspects of CHTTs. Patients highlighted their preference for the rapid accessibility of CHTT services, serving as a better alternative to inpatient admissions. Patients also appreciated the positive traits of CHTT staff and valued the shared decision-making during treatment. Patients emphasised the importance of feeling heard, safe, and well-supported during treatment, which contributed to developing strong therapeutic relationships with CHTT staff. Despite these positive experiences, the current review also identified negative experiences of CHTTs. These included staff timekeeping, impersonal and generic care, inconsistent personnel and challenges with transitioning to other services. These aspects hindered the formation of a trusting therapeutic relationship, which ultimately lead to poor communication between patients and staff. Moreover, some participants preferred inpatient services to CHTTs, as it is a separate environment compared to their own homes. Allowing patients to choose and control their care where possible suggests the need for efficient collaboration and transition planning between CHTTs and inpatient wards. This collaboration can facilitate smooth transitions from CHTTs to inpatient care depending on patient needs. These findings triangulate with similar systematic reviews and quantitative research that reported similar results [11, 12, 31, 32, 33, 34], despite utilising different methodologies. These studies reported comparable findings, such as seeing CHTTs as a better alternate for inpatient care, the importance of having rapid access to services, fostering strong therapeutic relationships and having consistent personnel during treatment. These studies also reported similar concerns regarding inconsistencies in service delivery, receiving contradicting advice, and patients were least satisfied with end of care communication [12, 31]. Interestingly, one study suggested that the commonly mentioned limitation of personnel inconsistencies may have unrecognised benefits. Specifically, providing various therapeutic approaches to patients during treatment, and preventing staff burnout [11]. Therefore, it is important to note that aspects that patients were least satisfied with may not necessarily be limitations to service implementation. Given the differing nature of CHTT implementation, the implementation of services are subject to the circumstances and governance of each CHTT or region [11]. Several quantitative studies examining patient satisfaction on CHTTs revealed an overall trend of positive experiences using CHTTs, however, not a statistically significant difference [35]. Additionally, another quantitative study examining the effectiveness of CHTTs found that 93% of patients reported some clinical improvements during their care [36]. These findings align with the themes identified in this review, highlighting positive experiences in accessing CHTTs.

However, it is important to note that previous studies and reviews have different study designs and inclusion criteria. Previous reviews examined patient experiences on CHTTs with a focus on different crisis intervention services (e.g. mobile crisis intervention services) or demographic groups (e.g. older populations). Some studies also incorporated quantitative data, randomised controlled trials, and published policy guidelines regarding CHTTs [12, 31, 32, 34] along with qualitative data. The data analysis method of previous systematic reviews differs from the current systematic review. Previous reviews utilised narrative syntheses and meta-analyses. This highlights the difference in the study designs between the current systematic review and previous reviews. The variations in study design may lead to a difference in results as the method of analysis varies and is difficult to compare.

### Strengths and limitations

The current systematic review stands out as the first to exclusively synthesise existing qualitative data on patient experiences of CHTTs. This review is registered on PROSPERO and adhered to the PRISMA guidelines on conducting systematic reviews, demonstrating rigorous methodology. The literature search was conducted on multiple databases, and search terms consisted of numerous subject headings and key words to gain a wider scope of existing clinical research. The screening was also completed with two independent reviewers. Moreover, the included studies were rated with high CASP scores, and the inclusion of patients with varying mental health diagnoses and contexts (e.g. perinatal women) further showcases the robustness of this review.

Nonetheless, the limitations also merit consideration. The total sample size of the included studies varies largely, ranging from a study with just one participant to a study with 117 participants. Having a small sample, especially with only one participant, may threaten the content validity [37] and generalisability as the accounts of a single individual may not fully and accurately represent the experiences of other patients. Therefore, conclusions should be interpreted with caution. Moreover, the variability of data analysis methods in the included studies is noteworthy. Additionally, the study settings of included studies were only based in two regions, with the seven studies conducted in the UK and the remaining three in Europe (two in Norway, one in Spain). There was also a lack of specification of patient’s individual characteristics (e.g. their age, ethnicity or mental health diagnoses) of included studies. Therefore, the results of these studies cannot be generalised to other regions or diverse populations. Moreover, the majority of participants included in the studies were mainly female patients, suggesting an unequal gender representation. Thus, the results cannot be generalised to male patients as their experiences may differ. Furthermore, the consistency of implementing CHTTs services across included studies is uncertain as they were conducted in diverse settings. Additionally, the duration of patients receiving treatment also varies in the included studies. Some patients had only received treatment for as little as two days [23], while others were no longer in treatment [16]. Patients that received a short-term treatment may not provide a comprehensive view of the service, and patients recalling their experiences after six months of treatment may lead to inaccuracies in recollection, which can lead to recall bias [38]. In relation to the search strategy, we only utilised Google Scholar to search for grey literature, and did not examine reference lists to find additional papers, which may have resulted in papers being missed. We also excluded eight studies due to the inaccessibility of full texts and should have attempted to contact the corresponding authors to retrieve them. Lastly, the current systematic review is also dependent on interpretations and quotes provided by the authors of the included studies. This approach can be limiting as it restricts the current authors to examine qualitative data in depth and form a comprehensive understanding of the unique nuances of patient experiences.

### Clinical implications and future research

**Table:** 

Key Recommendations from Current Study	QN-CRHTT Guidelines
Include additional nuances in service delivery (e.g. going on walks with patients) could be helpful in improving quality of care.	Staff are trained in aspects including physical assessments, risk assessments and delivering crisis interventions.
Training staff for specific contexts (e.g. perinatal women) and establishing tailored interventions for various demographics could enhance therapeutic relationships between patients and CHTT staff.	There is a specific care plan for perinatal women, but focuses on assessments, medication prescription, and the referral to specialists in perinatal units.
Incorporate and emphasise the importance of staff punctuality and consistency in the QN-CRHTT to refine service delivery.	Staff punctuality and consistency in service delivery are not mentioned.

Despite these limitations, the current review sheds light on patient insights on CHTTs, and aspects that help and hinder patients’ crisis recovery within CHTTs. The inclusion of patient input can provide valuable recommendations and guidance for enhancing service governance and quality of care [14]. The conclusions derived from this review highlights the significance of cultivating strong therapeutic relationships between patients and staff, deliver well-tailored services with consistent personnel, and provide seamless transitions to end of care or other services. The findings of the current review also demonstrate the difference in service implementation in regions such as the UK and Norway. For instance, in Norway, CHTT staff taking patients on a walk is a gesture that is highly appreciated in patients. Such aspects of care were not referred to in UK studies, suggesting a difference in service implementation between the two regions. These additional nuances of service delivery seen in Norwegian CHTTs can act as a clinical guidance for services in other regions to incorporate similar aspects to improve quality of care. However, further research is needed within the context of CHTTs. Future research calls for more larger-scaled qualitative research in various geographical regions to gain a better understanding on the similarities and discrepancies in service delivery and patient experiences on CHTTs.

In relation to current guidance outlined in the QN-CRHTT [39], the policy suggests CHTT staff to be trained in various aspects including physical assessments, risk assessments and delivering crisis interventions (e.g. psychosocial interventions). However, emphasising a focus on training for specific contexts and demographics can be a useful approach in delivering well-tailored services. For instance, in the context of perinatal women, the QN-CRHTT consists of a specific care plan for perinatal women, but with a focus on assessments, medication prescription, and the referral to specialists in perinatal units [39]. Instead of referring patients to a perinatal service, equipping CHTT staff with tailored interventions for patients in perinatal periods could strengthen therapeutic relationships. Understanding mental health crises in a perinatal context and providing tailored treatment to mothers is essential to improving trusting relations between patients and CHTT staff. Furthermore, concerns such as staff punctuality and consistency raised by patients are not currently addressed in the QN-CRHTT guidelines. Incorporating patient feedback such as improvements in staff punctuality and consistent continuity of care into service policies can refine service delivery and patient satisfaction.

In sum, the current review highlights the importance of patient input on CHTTs and the need for further qualitative research. With growing interest and investments in crisis services, considering patient recommendations can be useful for service planners or policymakers of CHTT teams to enhance and expand service resources and patient experience during a time of crisis.

## Electronic supplementary material

Below is the link to the electronic supplementary material.

Supplementary Material

## Data Availability

No datasets were generated or analysed during the current study.

## Declarations

### Transparency declaration

The authors can confirm that this is an honest, accurate, and transparent account of the study being reported; that no important aspects of the study have been omitted; and that any discrepancies from the study as planned (and, if relevant, registered) have been explained.
