# Supplementary material for: Patient experiences of crisis home treatment teams: a systematic review and thematic synthesis
Source: Soc Psychiatry Psychiatr Epidemiol. 2025 Feb 17;60(9):2035–47. doi: 10.1007/s00127-025-02830-6 (PMC12378471; doi:10.1007/s00127-025-02830-6)
Supplement: Supplementary file 1 — Supplementary Material [file 127_2025_2830_MOESM1_ESM.docx]

**Appendix 1 – Search strategy for each database**

**Medline**

| **#** | **Query** | **Results from 27 Jun 2023** |
| --- | --- | --- |
| 1 | patients/ or outpatients/ | 45,015 |
| 2 | attitude/ or patient compliance/ or patient dropouts/ or patient participation/ or patient satisfaction/ or patient preference/ or respect/ | 243,412 |
| 3 | 1 and 2 | 3,916 |
| 4 | ((Service user* or client* or patient* or out patient* or consumer* or client* or "user* of service*") adj3 (experienc* or satisf* or access* or quality* or consisten* or feedback* or opinion* or feeling* or perspectiv* or views* or subjective)).ti,ab. | 386,393 |
| 5 | 3 or 4 | 389,006 |
| 6 | Crisis Intervention/ | 6,228 |
| 7 | Home Care Services/ | 36,326 |
| 8 | home treatment team*.ti,ab. | 81 |
| 9 | intensive treatment team*.ti,ab. | 4 |
| 10 | mental health service*.ti,ab. | 25,169 |
| 11 | mental health team*.ti,ab. | 1,060 |
| 12 | crisis service*.ti,ab. | 271 |
| 13 | crisis care.ti,ab. | 172 |
| 14 | crisis resolution*.ti,ab. | 216 |
| 15 | crisis intervention*.ti,ab. | 1,912 |
| 16 | crisis team*.ti,ab. | 168 |
| 17 | home based treatment team*.ti,ab. | 2 |
| 18 | or/6-17 | 69,184 |
| 19 | (interview* or questionnaire* or survey*).ti,ab. | 1,660,228 |
| 20 | (focus group* or qualitative or ethnograph* or fieldwork or "field work" or "key informant" or "mixed method*").ti,ab. | 378,387 |
| 21 | Interviews as Topic/ | 66,816 |
| 22 | Focus Groups/ | 35,789 |
| 23 | Narration/ | 10,138 |
| 24 | qualitative research/ | 81,770 |
| 25 | 19 or 20 or 21 or 22 or 23 or 24 | 1,889,718 |
| 26 | 5 and 18 and 25 | 1,991 |

**Embase**

| **#** | **Query** | **Results from 27 Jun 2023** |
| --- | --- | --- |
| 1 | patient/ or outpatient/ | 1,614,347 |
| 2 | consumer/ | 59,308 |
| 3 | 1 or 2 | 1,670,678 |
| 4 | personal experience/ | 62,682 |
| 5 | attitude/ or consumer attitude/ or patient attitude/ or respect/ | 157,179 |
| 6 | patient attendance/ or patient compliance/ or patient dropout/ or patient engagement/ or patient participation/ or patient preference/ or patient satisfaction/ | 372,253 |
| 7 | 4 or 5 or 6 | 568,989 |
| 8 | 3 and 7 | 34,830 |
| 9 | ((Service user* or client* or patient* or out patient* or consumer* or client* or "user* of service*") adj3 (experienc* or satisf* or access* or quality* or consisten* or feedback* or opinion* or feeling* or perspectiv* or views* or subjective)).ti,ab. | 624,516 |
| 10 | 8 or 9 | 643,970 |
| 11 | crisis intervention/ | 6,649 |
| 12 | home care/ or home mental health care/ | 70,233 |
| 13 | home treatment team*.ti,ab. | 151 |
| 14 | intensive treatment team*.ti,ab. | 5 |
| 15 | mental health service*.ti,ab. | 31,002 |
| 16 | mental health team*.ti,ab. | 1,651 |
| 17 | crisis service*.ti,ab. | 348 |
| 18 | crisis care.ti,ab. | 217 |
| 19 | crisis resolution*.ti,ab. | 292 |
| 20 | crisis intervention*.ti,ab. | 2,424 |
| 21 | crisis team*.ti,ab. | 239 |
| 22 | home based treatment team*.ti,ab. | 3 |
| 23 | or/11-22 | 109,585 |
| 24 | (interview* or questionnaire* or survey*).ti,ab. | 2,222,806 |
| 25 | (focus group* or qualitative or ethnograph* or fieldwork or "field work" or "key informant" or "mixed method*").ti,ab. | 471,207 |
| 26 | exp *interview/ | 20,243 |
| 27 | narrative/ or storytelling/ | 26,434 |
| 28 | qualitative research/ | 117,102 |
| 29 | or/24-28 | 2,508,354 |
| 30 | 10 and 23 and 29 | 3,443 |

**PsychINFO**

| **#** | **Query** | **Results from 27 Jun 2023** |
| --- | --- | --- |
| 1 | patients/ or outpatients/ | 35,443 |
| 2 | client attitudes/ or client satisfaction/ | 25,265 |
| 3 | consumer attitudes/ or consumer satisfaction/ or consumer research/ or consumer surveys/ or "quality of services"/ | 30,984 |
| 4 | attitudes/ or adult attitudes/ or preferences/ | 65,204 |
| 5 | treatment compliance/ or treatment dropouts/ | 20,041 |
| 6 | client participation/ | 3,145 |
| 7 | respect/ | 1,580 |
| 8 | or/2-7 | 141,673 |
| 9 | 1 and 8 | 2,275 |
| 10 | ((Service user* or client* or patient* or out patient* or consumer* or client* or "user* of service*") adj3 (experienc* or satisf* or access* or quality* or consisten* or feedback* or opinion* or feeling* or perspectiv* or views* or subjective)).ti,ab. | 74,268 |
| 11 | 9 or 10 | 75,905 |
| 12 | crisis intervention/ or suicide prevention/ | 10,418 |
| 13 | crisis intervention services/ | 1,718 |
| 14 | home care/ | 7,585 |
| 15 | home treatment team*.ti,ab. | 102 |
| 16 | intensive treatment team*.ti,ab. | 8 |
| 17 | mental health service*.ti,ab. | 31,443 |
| 18 | mental health team*.ti,ab. | 1,255 |
| 19 | crisis service*.ti,ab. | 393 |
| 20 | crisis care.ti,ab. | 127 |
| 21 | crisis resolution*.ti,ab. | 269 |
| 22 | crisis intervention*.ti,ab. | 3,872 |
| 23 | crisis team*.ti,ab. | 242 |
| 24 | home based treatment team*.ti,ab. | 5 |
| 25 | or/12-24 | 52,846 |
| 26 | (interview* or questionnaire* or survey*).ti,ab. | 928,055 |
| 27 | (focus group* or qualitative or ethnograph* or fieldwork or "field work" or "key informant" or "mixed method*").ti,ab. | 281,353 |
| 28 | interviews/ or focus group interview/ or semi-structured interview/ | 14,222 |
| 29 | exp qualitative methods/ or mixed methods research/ | 21,524 |
| 30 | narratives/ or storytelling/ | 29,398 |
| 31 | or/26-30 | 1,076,732 |
| 32 | 11 and 25 and 31 | 1,526 |

**CINAHL**

| **#** | **Query** | **Results from 27 Jun 2023** |
| --- | --- | --- |
| S33 | S13 AND S26 AND S32 | 1,945 |
| S32 | S27 OR S28 OR S29 OR S30 OR S31 | Display |
| S31 | (MH "Qualitative Studies") OR (MH "Ethnographic Research") | Display |
| S30 | (MH "Focus Groups") | Display |
| S29 | (MH "Interviews+") OR (MH "Narratives") OR (MH "Surveys") OR (MH "Self Report") | Display |
| S28 | TI ( ("focus group*" or qualitative or ethnograph* or fieldwork or "field work" or "key informant" or "mixed method*") ) OR AB ( ("focus group*" or qualitative or ethnograph* or fieldwork or "field work" or "key informant" or "mixed method*") ) | Display |
| S27 | TI ( interview* or questionnaire* or survey* ) OR AB ( interview* or questionnaire* or survey* ) | Display |
| S26 | S14 OR S15 OR OR S17 OR S18 S19 OR S20 OR OR S22 OR S23 OR S24 OR S25 | Display |
| S25 | TI "home based treatment team*" OR AB "home based treatment team*" | Display |
| S24 | TI "crisis team*" OR AB "crisis team*" | Display |
| S23 | TI "crisis intervention*" OR AB "crisis intervention*" | Display |
| S22 | TI "crisis resolution*" OR AB "crisis resolution*" | Display |
| S21 | TI "crisis care" OR AB "crisis care" | Display |
| S20 | TI "crisis service*" OR AB "crisis service*" | Display |
| S19 | TI "mental health team*" OR AB "mental health team*" | Display |
| S18 | TI "mental health service*" OR AB "mental health service*" | Display |
| S17 | TI "intensive treatment team*" OR AB "intensive treatment team*" | Display |
| S16 | TI "home treatment team*" OR AB "home treatment team*" | Display |
| S15 | (MH "Home Health Care") OR (MH "Psychiatric Home Care") | Display |
| S14 | (MH "Crisis Intervention") | Display |
| S13 | S11 OR S12 | Display |
| S12 | TI ( ("Service user*" or client* or patient* or "out patient*" or consumer* or client* or "user* of service*") N3 (experienc* or satisf* or access* or quality* or consisten* or feedback* or opinion* or feeling* or perspectiv* or views* or subjective) ) OR AB ( ("Service user*" or client* or patient* or "out patient*" or consumer* or client* or "user* of service*") N3 (experienc* or satisf* or access* or quality* or consisten* or feedback* or opinion* or feeling* or perspectiv* or views* or subjective) ) | Display |
| S11 | S9 AND S10 | Display |
| S10 | S3 OR S4 OR S5 OR S6 OR S7 OR S8 | Display |
| S9 | S1 OR S2 | Display |
| S8 | (MH "Respect") | Display |
| S7 | (MH "Consumer Participation") | Display |
| S6 | (MH "Patient Dropouts") | Display |
| S5 | (MH "Patient Compliance") | Display |
| S4 | (MH "Consumer Satisfaction") OR (MH "Patient Satisfaction") OR (MH "Patient Preference") | Display |
| S3 | (MH "Attitude") OR (MH "Consumer Attitudes") OR (MH "Patient Attitudes") | Display |
| S2 | (MH "Consumers") | Display |
| S1 | (MH "Patients") OR (MH "Outpatients") | Display |

| Appendix 2 – CASP ratings | |
| --- | --- |
| CASP Statement | Carpenter & Tracy (2015): Home Treatment Teams: What should they do? A qualitative study on patient opinions |
| Was there a clear statement of the aims of the research? | Yes |
| Is a qualitative methodology appropriate? | Yes |
| Was the research design appropriate to address the aims of the research? | Yes |
| Was the recruitment strategy appropriate to the aims of the research? | Yes |
| Was the data collected in a way that addressed the research issue? | Yes |
| Has the relationship between researcher and participants been adequately considered? | Yes |
| Have ethical issues been taken into consideration? | Yes |
| Was the data analysis sufficiently rigorous? | Yes |
| Is there a clear statement of findings? | Yes |
| How valuable is the research? | The research presents valuable findings through thematic analysis and detailed discussions that align with existing literature on CHTTs. |

| CASP Statement | Giménez-Díez, Alía, Jiménez, et al. (2019): Treating mental health crises at home: Patient satisfaction with home nursing care |
| --- | --- |
| Was there a clear statement of the aims of the research? | Yes |
| Is a qualitative methodology appropriate? | Yes |
| Was the research design appropriate to address the aims of the research? | Yes |
| Was the recruitment strategy appropriate to the aims of the research? | Yes |
| Was the data collected in a way that addressed the research issue? | Yes |
| Has the relationship between researcher and participants been adequately considered? | Yes |
| Have ethical issues been taken into consideration? | Yes |
| Was the data analysis sufficiently rigorous? | Yes |
| Is there a clear statement of findings? | Yes |
| How valuable is the research? | The research presents valuable qualitative findings through thematic analysis and detailed discussions that align with existing literature on CHTTs. |

| CASP Statement | Hopkins & Niemiec (2007): Mental health crisis at home: service user perspectives on what helps and what hinders |
| --- | --- |
| Was there a clear statement of the aims of the research? | Yes |
| Is a qualitative methodology appropriate? | Yes |
| Was the research design appropriate to address the aims of the research? | Yes |
| Was the recruitment strategy appropriate to the aims of the research? | Yes |
| Was the data collected in a way that addressed the research issue? | Yes |
| Has the relationship between researcher and participants been adequately considered? | Yes |
| Have ethical issues been taken into consideration? | Yes |
| Was the data analysis sufficiently rigorous? | Yes |
| Is there a clear statement of findings? | Yes |
| How valuable is the research? | The research presents valuable qualitative findings through thematic analysis and detailed discussions that align with existing literature on CHTTs. |

| CASP Statement | Hubbeling & Bertram (2014): Hope, happiness and home treatment: a study into patient satisfaction with being treated at home |
| --- | --- |
| Was there a clear statement of the aims of the research? | Yes |
| Is a qualitative methodology appropriate? | Yes |
| Was the research design appropriate to address the aims of the research? | Yes |
| Was the recruitment strategy appropriate to the aims of the research? | No |
| Was the data collected in a way that addressed the research issue? | Yes |
| Has the relationship between researcher and participants been adequately considered? | Yes |
| Have ethical issues been taken into consideration? | No |
| Was the data analysis sufficiently rigorous? | No |
| Is there a clear statement of findings? | Yes |
| How valuable is the research? | The research presents valuable quantitative and qualitative findings, but the process of recruitment, and ethical considerations need to be discussed further. |

| CASP Statement | Karlsson, Borg & Kim (2008): From good intentions to real life: introducing crisis resolution teams in Norway |
| --- | --- |
| Was there a clear statement of the aims of the research? | Yes |
| Is a qualitative methodology appropriate? | Yes |
| Was the research design appropriate to address the aims of the research? | Yes |
| Was the recruitment strategy appropriate to the aims of the research? | Yes |
| Was the data collected in a way that addressed the research issue? | Yes |
| Has the relationship between researcher and participants been adequately considered? | No |
| Have ethical issues been taken into consideration? | No |
| Was the data analysis sufficiently rigorous? | Yes |
| Is there a clear statement of findings? | Yes |
| How valuable is the research? | The research presents valuable qualitative findings through thematic analysis and detailed discussions that align with existing literature on CHTTs. However, the relationship between researcher and participants were not specified. |

| CASP Statement | Khalifeh, Murgatroyd, Freeman et al. (2009): Home Treatment as an Alternative to Hospital Admission for Mothers in a Mental Health Crisis: A Qualitative Study |
| --- | --- |
| Was there a clear statement of the aims of the research? | Yes |
| Is a qualitative methodology appropriate? | Yes |
| Was the research design appropriate to address the aims of the research? | Yes |
| Was the recruitment strategy appropriate to the aims of the research? | Yes |
| Was the data collected in a way that addressed the research issue? | Yes |
| Has the relationship between researcher and participants been adequately considered? | Yes |
| Have ethical issues been taken into consideration? | Yes |
| Was the data analysis sufficiently rigorous? | Yes |
| Is there a clear statement of findings? | Yes |
| How valuable is the research? | The research presents valuable qualitative findings through content analysis and biases were considered well. |

| CASP Statement | Klevan, Karlsson & Ruud (2017): “At the extremities of life” – Service user experiences of helpful help in mental health crises |
| --- | --- |
| Was there a clear statement of the aims of the research? | Yes |
| Is a qualitative methodology appropriate? | Yes |
| Was the research design appropriate to address the aims of the research? | Yes |
| Was the recruitment strategy appropriate to the aims of the research? | Yes |
| Was the data collected in a way that addressed the research issue? | Yes |
| Has the relationship between researcher and participants been adequately considered? | No |
| Have ethical issues been taken into consideration? | Yes |
| Was the data analysis sufficiently rigorous? | Yes |
| Is there a clear statement of findings? | Yes |
| How valuable is the research? | The research presents valuable qualitative findings through a hermeneutic phenomenological approach and results were discussed in great detail. |

| CASP Statement | Morant, Lloyd-Evans, Lamb et al. (2017): Crisis resolution and home treatment: stakeholders’ views on critical ingredients and implementation in England |
| --- | --- |
| Was there a clear statement of the aims of the research? | Yes |
| Is a qualitative methodology appropriate? | Yes |
| Was the research design appropriate to address the aims of the research? | Yes |
| Was the recruitment strategy appropriate to the aims of the research? | Yes |
| Was the data collected in a way that addressed the research issue? | Yes |
| Has the relationship between researcher and participants been adequately considered? | Yes |
| Have ethical issues been taken into consideration? | Yes |
| Was the data analysis sufficiently rigorous? | Yes |
| Is there a clear statement of findings? | Yes |
| How valuable is the research? | The research presents valuable qualitative findings through thematic analysis. Findings also provide important service-users’ and carers’ experiences in multiple CHTT sites across England. |

| CASP Statement | Nelson & Ashman (2016): ‘Dale’: an interpretative phenomenological analysis of a service user’s experience with a crisis resolution/home treatment team in the United Kingdom |
| --- | --- |
| Was there a clear statement of the aims of the research? | Yes |
| Is a qualitative methodology appropriate? | Yes |
| Was the research design appropriate to address the aims of the research? | Yes |
| Was the recruitment strategy appropriate to the aims of the research? | Can’t Tell |
| Was the data collected in a way that addressed the research issue? | Yes |
| Has the relationship between researcher and participants been adequately considered? | No |
| Have ethical issues been taken into consideration? | Yes |
| Was the data analysis sufficiently rigorous? | Yes |
| Is there a clear statement of findings? | Yes |
| How valuable is the research? | This case study provided detailed findings through an interpretative phenomenological approach. However, as this is a case study on one participant, biases need to be considered. |
| CASP Statement | **Rubio, Taylor, Morant & Johnson (2021):** Experiences of intensive home treatment for a mental health crisis during the perinatal period: A UK qualitative study |
| Was there a clear statement of the aims of the research? | Yes |
| Is a qualitative methodology appropriate? | Yes |
| Was the research design appropriate to address the aims of the research? | Yes |
| Was the recruitment strategy appropriate to the aims of the research? | Yes |
| Was the data collected in a way that addressed the research issue? | Yes |
| Has the relationship between researcher and participants been adequately considered? | Yes |
| Have ethical issues been taken into consideration? | Yes |
| Was the data analysis sufficiently rigorous? | Yes |
| Is there a clear statement of findings? | Yes |
| How valuable is the research? | This study provided detailed findings that are valuable to existing research on perinatal women’s experiences on CHTT services. |
